# *Dens Invaginatus*: A Comprehensive Review of Classification and Clinical Approaches

**DOI:** 10.3390/medicina61071281

**Published:** 2025-07-16

**Authors:** Abayomi O. Baruwa, Craig Anderson, Adam Monroe, Flávia Cracel Nogueira, Luís Corte-Real, Jorge N. R. Martins

**Affiliations:** 1 Department of Clinical Sciences, College of Dentistry, Ajman University, Ajman 346, United Arab Emirates; 2 Private Practice, Biloxi, MS 39532, USA; 3 Private Practice, Vista, CA 92083, USA; 4 Private Practice, 4700-262 Braga, Portugal; 5 Department of Endodontics, Cooperativa de Ensino Superior Politécnico e Universitário (CESPU), 4585-116 Gandra, Portugal; drcortereal@gmail.com; 6 Faculdade de Medicina Dentária, Universidade de Lisboa, 1600-277 Lisboa, Portugal; 7 LIBPhys-FCT UID/FIS/04559/2013, 1600-277 Lisboa, Portugal; 8 Grupo de Investigação em Bioquimica e Biologia Oral (GIBBO), Unidade de Investigação em Ciências Orais e Biomédicas (UICOB), 1600-277 Lisboa, Portugal; 9 Centro de Estudos de Medicina Dentária Baseada na Evidência (CEMDBE), 1600-277 Lisboa, Portugal

**Keywords:** *dens invaginatus*, endodontics, dental anomaly, root canal therapy, treatment

## Abstract

*Dens invaginatus* is a developmental dental anomaly characterized by the infolding of the enamel organ into the dental papilla during early odontogenesis. This process leads to a broad spectrum of anatomical variations, ranging from minor enamel-lined pits confined to the crown to deep invaginations extending through the root, occasionally communicating with periodontal or periapical tissues. The internal complexity of affected teeth presents diagnostic and therapeutic challenges, particularly in severe forms that mimic root canal systems or are associated with pulpal or periapical pathology. Maxillary lateral incisors are most frequently affected, likely due to their unique developmental timeline and morphological susceptibility. Although various classification systems have been proposed, Oehlers’ classification remains the most clinically relevant due to its simplicity and correlation with treatment complexity. Recent advances in diagnostic imaging, especially cone beam computed tomography (CBCT), have revolutionized the identification and classification of these anomalies. CBCT-based adaptations of Oehlers’ classification allow for the precise assessment of invagination extent and pulpal involvement, facilitating improved treatment planning. Contemporary therapeutic strategies now include calcium-silicate-based cement sealing materials, endodontic microsurgery for inaccessible anatomy, and regenerative endodontic procedures for immature teeth with necrotic pulps. Emerging developments in artificial intelligence, genetic research, and tissue engineering promise to further refine diagnostic capabilities and treatment options. Early detection remains critical to prevent complications such as pulpal necrosis or apical disease. A multidisciplinary, image-guided, and patient-centered approach is essential for optimizing clinical outcomes in cases of *dens invaginatus*.

## 1. Introduction

*Dens invaginatus* is a well-documented developmental dental anomaly defined by an invagination of the enamel organ into the dental papilla during the morphodifferentiation stage, prior to tissue calcification [1]. This aberrant developmental process leads to highly variable and complex internal tooth anatomy, which often complicates both diagnosis and endodontic treatment. The extent of the invagination varies significantly, ranging from minor forms confined to the crown to more extensive presentations that penetrate the root and may reach the apical foramen [2].

The anomaly was first described in humans during the 19th century, with early terminology reflecting diverse interpretations of its pathogenesis and morphology [3]. Historically, various terms such as “tooth within a tooth,” “invaginated odontome,” “dilated composite odontome,” and “gestant anomaly” were employed to describe *dens invaginatus* [4]. The term *dens in dente* was introduced by Busch in 1897 based on its radiographic appearance [3]. However, the term *dens invaginatus* has gained preference in the contemporary literature, as it more accurately denotes the invagination of external dental tissues, primarily enamel, into the inner structure of the developing tooth, resulting in the formation of an atypical internal cavity.

The exact etiology of *dens invaginatus* remains uncertain, and multiple theories have been proposed. One of the most widely accepted is the growth slowing theory, which posits that a localized failure in the development of the internal enamel epithelium, amidst the ongoing proliferation of surrounding tissues, leads to the inward folding of the enamel organ [3,5]. Alternatively, some authors suggest that accelerated proliferation of the internal enamel epithelium may be responsible [6,7]. Additional hypotheses include fusion of adjacent tooth germs, traumatic injury during odontogenesis, or localized infection [2,6]. Emerging evidence also points to a genetic predisposition, particularly in cases with bilateral or familial involvement [7]. *Dens invaginatus* has been observed more frequently in individuals with other developmental anomalies such as taurodontism and microdontia, suggesting a potential shared genetic etiology. In a classical study by Grahnen et al. [8], 43% of cases had a positive family history, with 32% of siblings were also affected. Furthermore, genetic syndromes associated with chromosomal anomalies have been linked to the presence of *dens invaginatus*, reinforcing the hypothesis of a genetic component [7,9,10].

Recent epidemiological data suggest that *dens invaginatus* may be more prevalent than previously thought. Although prevalence rates vary considerably across studies, it is generally recognized as the second most common dental developmental anomaly after dental agenesis. Reported prevalence ranges from 0.25% to 10.0% in the general population [11], with rates as high as 26.1% reported in specific subpopulations, such as orthodontic patients [12]. These discrepancies likely reflect differences in study design, diagnostic methodology, and sample characteristics. Traditional radiographic techniques may underestimate prevalence, especially in mild or coronal forms. Conversely, cone beam computed tomography (CBCT) has significantly improved detection by enabling the three-dimensional visualization of invaginations, particularly those extending into the root [13]. From an anatomical perspective, the maxillary lateral incisor is by far the most frequently affected tooth [3]. Other teeth such as maxillary central incisors, canines, premolars, and molars are less commonly involved, while occurrences in the mandibular dentition are rare [7,14]. The susceptibility of the lateral incisor may be attributed to its embryological development, which occurs in proximity to other tooth germs, potentially exposing it to perturbations during morphogenesis [15]. Bilateral or symmetrical occurrences have been reported in up to 43% of cases, although some authors caution that such presentations should be interpreted critically [7].

This review aims to provide an updated synthesis of the classification systems, clinical presentation, diagnostic strategies, and current management approaches for *dens invaginatus*, with a focus on integrating recent advances in imaging, materials, and treatment protocols to guide clinical decision-making.

## 2. Evolution of *Dens Invaginatus* Classifications

Historically, multiple classification systems for *dens invaginatus* have been proposed, with ongoing debate spanning several decades. Table 1 provides a comparative summary of the key features, relevance, and limitations of the various classification systems for *dens invaginatus.*

The earliest attempt was made by Hallet in 1953 [16], who classified the condition based on clinical and radiographic observations, particularly focusing on palatal invaginations of maxillary incisors. Hallet’s work laid the foundation for the widely accepted classification proposed by Oehlers in 1957 [17], which gained prominence due to its simplicity and clinical relevance. In 1964, Ulmansky and Hermel [18] introduced a classification that emphasized morphological, developmental, and histological variations. Their approach helped to define the broad spectrum of the anomaly, ranging from minor enamel-lined invaginations to gross anatomical malformations, thereby linking clinical presentation with underlying histopathology. Approximately eight years later, Schulze and Brand (1972) [19] further expanded the classification scope by identifying 12 distinct morphological types based on clinical and radiographic criteria. These descriptions considered the extent and shape of the invagination, its communication with the pulp or periodontal ligament, the number and direction of invaginations, and their impact on surrounding tooth structures. However, due to its complexity and limited practicality in clinical settings, this classification was seldom adopted in everyday practice. To address these limitations, Vincent-Townend (1974) [20] proposed a simplified version of the Schulze and Brand model. His classification grouped the 12 morphological types into a more concise structure that retained sufficient detail for clinical relevance while improving usability [11]. With the advent of cone beam computed tomography (CBCT), new classification systems have emerged [21,22,23,24]. The 3D capabilities of CBCT enable the precise visualization of the invagination’s extent and its communication with the pulp or periapical tissues, thereby enhancing diagnostic accuracy and guiding treatment planning [13]. A notable advancement in this regard was the CBCT-based classification system developed by Ahmed and Dummer in 2018 [21]. Building on Oehlers’ framework, they introduced a standardized anatomical coding scheme applicable to a wide range of root canal anomalies. This system incorporates variables such as tooth number, number of roots, canal configuration, and presence of anomalies, with each anomaly represented by a specific code letter. This approach improves anatomical precision, facilitates case documentation, and supports consistent clinical application.

## 3. Overview of *Dens Invaginatus* Classification

Considering the historical context previously outlined, the classifications below are presented in greater detail to illustrate the criteria, scope, and clinical relevance of each system.

Hallet’s (1953) [16]

This morphology is classified into four types based on the location and extent of the invagination:

- Type I: A small, enamel-lined invagination confined to the crown that does not extend beyond the cementoenamel junction (CEJ).
- Type II: An invagination that extends beyond the CEJ into the root but remains a blind sac. It may or may not communicate with the pulp.
- Type III: An invagination that penetrates deeply into the root and may form a second foramen, potentially communicating with the periapical tissues. There is usually no direct communication with the pulp, although it is often associated with periapical lesions despite the pulp remaining vital.
- Type IV: A complex or dilated invagination in which the entire tooth structure is distorted. This type may resemble a dilated composite odontoma.

Oehlers (1957) [17]

This classification system divides *dens invaginatus* into three types based on the depth and extent of the invagination:

- Type I: The invagination is confined to the crown and does not extend beyond the CEJ.
- Type II: The invagination extends beyond the CEJ and may involve the root, but it remains a blind sac. It may or may not communicate with the pulp.
- Type III: The invagination penetrates through the root and communicates with the periodontal ligament, laterally in Type IIIa and apically in Type IIIb, without direct communication with the pulp chamber.

Ulmansky & Hermel (1964) [18]

This classification focuses on the morphological and developmental variations in invaginations rather than relying solely on radiographic appearance. Unlike Oehlers’ system, it does not define types in a numbered format. Instead, it highlights the origin of the invagination, typically arising from the lingual pit or cingulum area, and considers the depth of its extension into the crown or root. The classification also emphasizes the identification of structural abnormalities, including

- Enamel-lined tracts;
- Communication with the pulp;
- Irregularities in the surrounding dentin;
- The presence of connective tissue or pulp-like remnants within the invagination.

Schulze & Brand (1972) [19]

This classification identifies twelve morphological types based on clinical and radiographic examination. Although not documented using a numerical system, the descriptions are structured around the following criteria:

- The extent and shape of the invagination;
- Communication with the pulp or periodontal ligament;
- The number and direction of the invaginations;
- The effect on surrounding tooth structures (e.g., dentin, enamel, and pulp).

Vincent Townend (1974) [20]

This classification includes types based on the following criteria:

- The extent of the invagination: Similar to Oehlers’ classification, ranging from coronal-only involvement to deep extensions into the root.
- Communication with the pulp and periodontium: Whether or not the invagination communicates with the pulp chamber or periapical tissues.
- Morphological complexity: Whether the invagination results in distorted crown or root morphology, as seen in dilated odontomes.
- Radiographic appearance: Assessment based on the visibility and characteristics of the invagination in radiographic imaging.

CBCT-Based Classifications [21,22,23,24]

Newer classification systems based on advanced imaging techniques have emerged to enhance the traditional Oehlers classification. These CBCT-based classifications provide a more precise description of invaginated structures and canal configurations, thereby supporting clinicians in documentation and treatment planning. One notable example is the root canal coding system developed by Ahmed and Dummer (2018) [21], which offers a standardized anatomical framework. Similarly, Gul et al. (2021) [22] proposed an additional Type IV for Oehlers’ classification, describing cases where the invagination extends into the pulp chamber beyond the amelodentinal junction and continues laterally and apically through a pseudoforamen. More recently, Wang et al. (2024) [23] expanded on these developments by categorizing *dens invaginatus* as either coronal or radicular. The radicular type was further divided into two main subtypes: (i) radicular cystoid invaginatus within an enlarged root; (ii) radicular grooves, which are further subdivided into three categories based on their extent [24].

## 4. Anatomic Distribution and Pathological Prevalence of *Dens Invaginatus* Types

In previously published prevalence studies, the most frequently observed type of *dens invaginatus* was Type I, according to Oehlers’ classification. Kirzioğlu and Ceyhan (2009) [25] found that Type I accounted for 94% of *dens invaginatus* cases, followed by 3% of Type II and 3% of Type III, among 300 affected patients in a sample of 2477 individuals (12% patient-level prevalence). In the study by Gündüz et al. (2013) [12], which assessed 4556 patients, Type I was also predominant, accounting for 70% of *dens invaginatus* cases. Capar et al. (2015) [26] corroborated these findings, observing that among the *dens invaginatus*-affected teeth identified through CBCT, 66% were Type I, 29% were Type II, and 5% were Type III. Collectively, these findings demonstrate that Type I *dens invaginatus* is by far the most common form, with Types II and III being considerably less frequent. In terms of prevalence among the global sample of examined teeth, *dens invaginatus* was found in approximately 0.65% of all teeth evaluated (Hamasha & Alomari, 2004) [27].

The risk of apical pathology increases with the severity of *dens invaginatus* type. Kirzioğlu and Ceyhan (2009) [25] reported that 33% of Type III cases were associated with apical pathosis, while 4% of Type II cases presented such pathology, and no significant pathology was found among Type I cases. Similarly, Capar et al. (2015) [26] noted that 100% of patients with Type III and 25% of those with Type II had apical pathosis, whereas no lesions were observed in Type I cases. Gündüz et al. (2013) [12] found that 88% of Type III and 8% of Type II cases had apical periodontitis, while only one lesion was found among all Type I cases. These findings are consistent with the observations by Alani and Bishop (2008) [11], who emphasized that the structural complexity and direct communication with periodontal structures in Type III invaginations predispose these teeth to periapical and pulpal disease. Hence, while Type I is the most prevalent, Type II and especially Type III are more clinically significant due to their higher pathological potential.

## 5. Clinical Features, Diagnostic Considerations, and Management

Generally, *dens invaginatus* may present with abnormal crown morphology, such as a peg-shaped, barrel-shaped, or dilated crown. Additional features can include exaggerated palatal cingula, talon cusps, incisal notching, or a deep foramen caecum. However, in many cases, there are no overt clinical signs, and the condition is discovered incidentally during routine dental radiographic examinations [7,28]. Radiographically, the anomaly typically appears as a well-defined radiolucent area within the crown or root, often bordered by an enamel-like radiopaque margin. The internal morphology can vary significantly, ranging from a simple groove or fissure to complex canal-like structures that may communicate with the pulp or periapical tissues [28]. Type I cases are more commonly encountered, while Types II and III are rarer and more difficult to identify [29]. Furthermore, the clinical presentation is variable, and the use of Oehlers’ classification helps to correlate the features of each type with treatment complexity and prognosis. Table 2 summarizes the risk of pulp and periapical involvement, the management strategies, and the prognosis.

Type I

Type I *dens invaginatus* frequently presents as a deep pit or groove on the palatal surface, resembling a foramen caecum or a deep fissure. Although the overall crown morphology may appear normal, subtle variations such as a prominent cingulum or the presence of a talon cusp may be observed. Enamel defects, including hypoplasia or localized discoloration, can also be present. Despite these anomalies, the pulp typically remains vital unless compromised by carious lesions. Radiographically, Type I appears as a loop-shaped radiolucency confined to the crown, surrounded by a radiopaque enamel-like lining [28].

**Figure 1:**
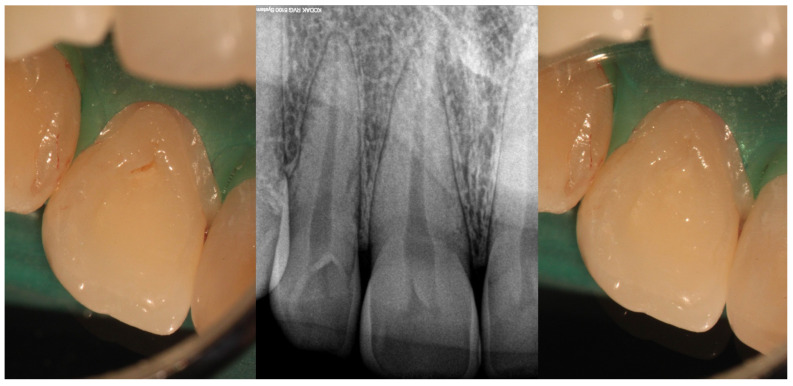
Clinical view of Oehlers Type I *dens invaginatus* (**left**) with its corresponding radiographic image (**center**) and after invagination sealing with flow composite (**right**) (courtesy of J.N.R.M.).

As for the clinical implications, Type I lesions pose minimal risk to the pulp in the absence of caries or bacterial ingress. However, their anatomical configuration can create niches that retain plaque and promote early decay. Preventive measures, such as sealing the palatal pit or groove with resin-based sealants, are generally sufficient and the prognosis is good with a very minimal risk of recurrence. Endodontic treatment is rarely required unless secondary infection occurs. Regular clinical and radiographic monitoring is advisable to detect early changes. Early intervention can prevent complications and preserve pulp vitality. Figure 1 illustrates the clinical presentation and management of an Oehlers Type I *dens invaginatus.*

Type II

Teeth with Type II *dens invaginatus* are often asymptomatic in the early stages but carry a higher risk of pulpal inflammation or necrosis. This increased risk is primarily due to a thin or incomplete enamel lining and the presence of micro-communications that facilitate bacterial ingress. Externally, the crown may exhibit morphological abnormalities such as a peg-shaped or barrel-shaped form, and deep palatal grooves are commonly noted. As the condition progresses, clinical signs may include tooth discoloration, pain, or the development of sinus tracts, particularly in cases where pulpal infection or necrosis has occurred. Radiographically, Type II typically appears as a tubular or sac-like radiolucency extending into the coronal third or middle portion of the root [30,31]. The invagination may appear to approximate or even communicate with the pulp space. Additionally, the surrounding dentin is often thin or irregular, further complicating the internal anatomy and increasing the risk of pulpal compromise.

**Figure 2:**
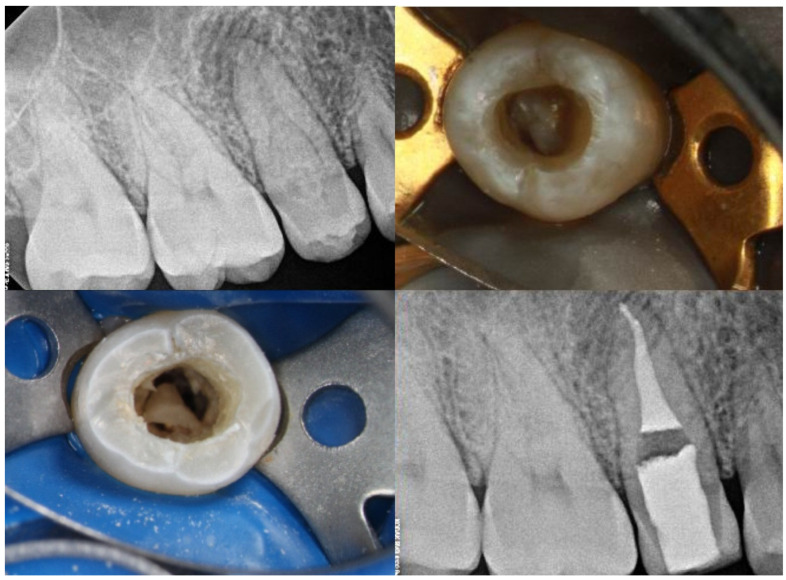
A representation of a rare Oehlers Type II *dens invaginatus* case observed in a maxillary second premolar, in which the invagination was removed during treatment (courtesy of C.A.).

**Figure 3:**
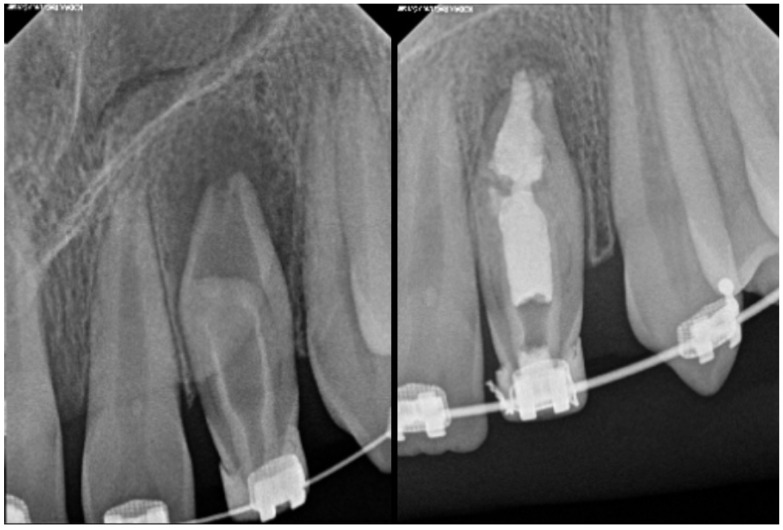
Radiographic features of Oehlers Type II *dens invaginatus* case in lateral incisor before (**left**) and after (**right**) treatment (courtesy of C.A.).

**Figure 4:**
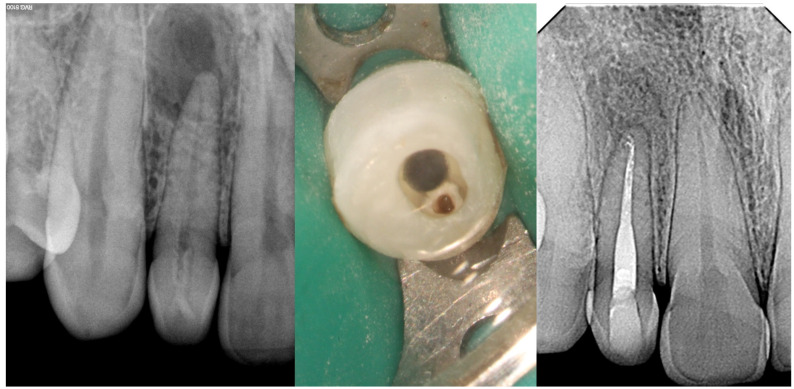
Representative images of an Oehlers Type II *dens invaginatus* in a lateral incisor before (**left**) and after (**right**) root canal therapy. The image in the center highlights the presence of enamel surrounding the lingual invagination (courtesy of J.N.R.M.).

As for the clinical implications, Type II lesions pose a significantly greater threat to pulpal health than Type I and therefore warrant careful evaluation. Cone beam computed tomography (CBCT) is strongly recommended to determine the true extent of the invagination and to identify any communication with the pulp [13]. Management should be guided by the vitality of the pulp. In cases without pulpal involvement, preventive sealing or minimally invasive restoration may be sufficient. However, when the pulp is compromised, vital pulp therapy or even conventional root canal treatment may be required depending on the pulp status. Early diagnosis and timely intervention are critical to preserving tooth vitality, minimizing complications, and improving long-term outcomes. The prognosis generally ranges from good to fair depending on the complexity and extent of the invagination with a moderate risk of recurrence. Figure 2, Figure 3 and Figure 4 illustrate the clinical presentation of Oehlers Type II *dens invaginatus.*

Type III

As with other forms of *dens invaginatus*, the crown morphology in Type IIIa is abnormal and may exhibit features such as a dilated, talon-like cusp. Despite the anatomical anomaly, the tooth often remains asymptomatic, even in the presence of lateral periodontal or periapical infections. Clinical signs, when present, may include localized swelling, deep isolated periodontal pockets, or sinus tract formation. In contrast, Type IIIb typically presents with symptoms of acute or chronic apical periodontitis, including periapical abscesses. Radiographically, Type IIIa appears as a radiolucent tract extending from the crown into the mid-root area, terminating laterally. This pattern may mimic a lateral periodontal or furcation lesion, making radiographic interpretation and correlation with clinical findings essential [28,32]. In Type IIIb, the radiolucent tract extends from the crown to the root apex and is frequently associated with a well-defined apical radiolucency. This configuration can mimic a pseudo-root canal or even give the appearance of a double-rooted structure, further complicating diagnosis [28,32,33].

**Figure 5:**
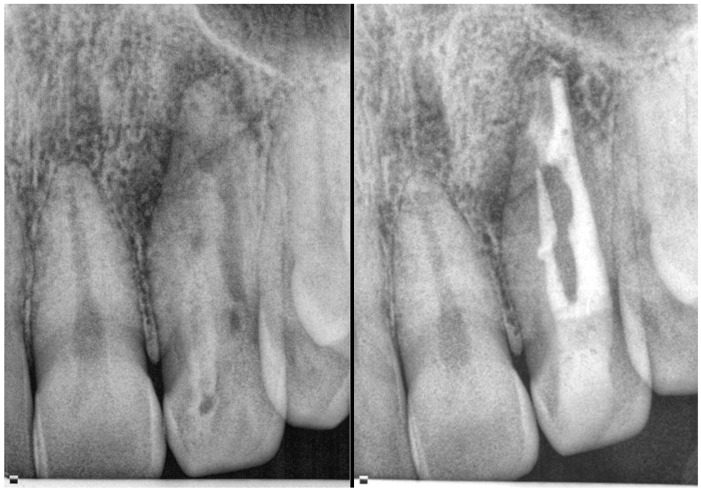
Illustration of Oehlers Type IIIa *dens invaginatus* case (courtesy of A.M.).

**Figure 6:**
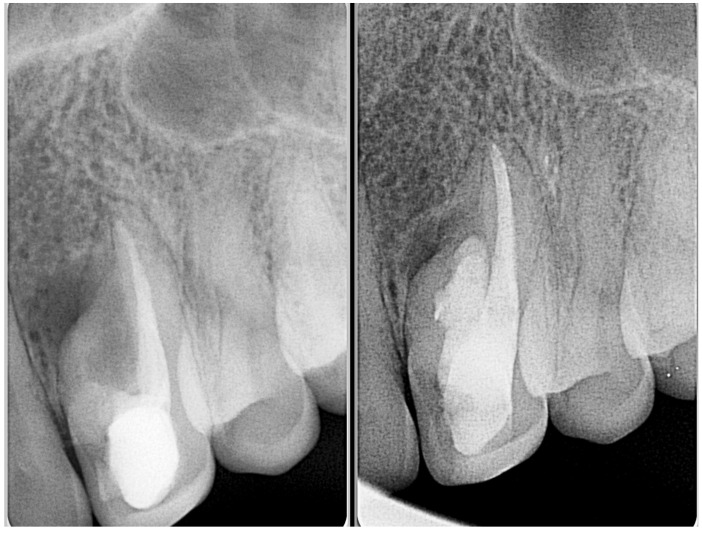
Radiographic images of Oehlers Type IIIa *dens invaginatus* case in maxillary canine (courtesy of L.C.R.).

**Figure 7:**
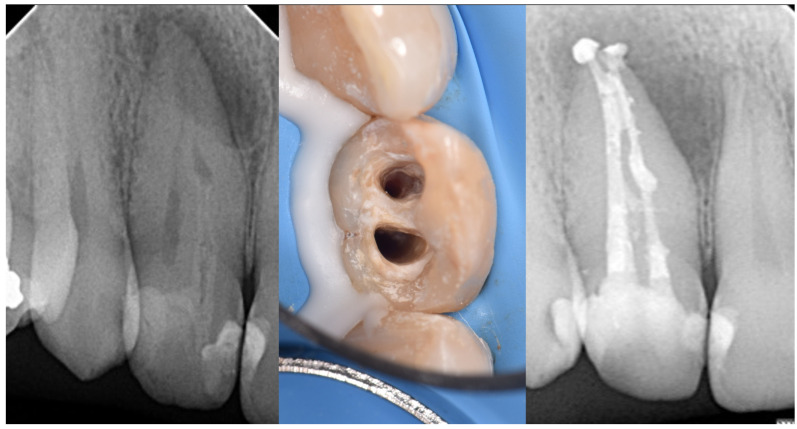
Radiographic images of Oehlers Type IIIb *dens invaginatus* case in maxillary lateral incisor (courtesy of F.C.N.).

As for the clinical implications, the diagnosis of Type III *dens invaginatus* is particularly challenging, as infection of the invaginated tract can occur independently of the main pulp tissue. Vitality testing may be misleading, especially when the primary root canal remains unaffected. CBCT is essential to determine the precise extent, course, and relationship of the invagination with the pulp and periapical tissues, thereby guiding appropriate treatment planning [13]. Management is often complex. In cases where the invagination is infected, but the main pulp remains vital, a conservative approach aiming to selectively treat the invagination may be considered. However, in most instances, surgical intervention is required to access and debride the invagination [28]. This is typically followed by orthograde or retrograde obturation using biocompatible materials. When there is concurrent periodontal involvement or anatomical complexity, a combined endodontic–periodontal approach may be necessary. Long-term follow-up is crucial due to the high risk of reinfection or lesion persistence. Figure 5, Figure 6 and Figure 7 illustrate the clinical presentation and radiographic characteristics of Oehlers Type IIIa and IIIb *dens invaginatus.*

## 6. Associated Dental Anomalies

*Dens invaginatus* is frequently associated with other dental anomalies, suggesting a shared developmental origin or an underlying genetic predisposition. Among the most common is the talon cusp, an accessory cusp typically arising from the cingulum area of maxillary anterior teeth, which is often observed in conjunction with Type I and II invaginations [34,35,36]. Peg-shaped maxillary lateral incisors are also a frequent finding and may reflect altered morphodifferentiation during crown development. In more severe cases, *dens invaginatus* can present as a dilated odontoma, a grossly malformed structure in which normal crown and root morphology is lost [15,17,37].

Enamel hypoplasia or hypomineralization has also been reported in association with *dens invaginatus*, often complicating oral hygiene and increasing susceptibility to early carious lesions [28]. Other anomalies, such as supernumerary teeth, taurodontism, microdontia, and macrodontia, have been documented as well, supporting the hypothesis that *dens invaginatus* may form part of a broader spectrum of dental developmental disturbances. Structural variations in the root, including short roots, dilacerations, and accessory roots or canals, are not uncommon and may significantly complicate endodontic treatment [10,38]. Additionally, teeth affected by *dens invaginatus* may exhibit pulpal calcifications or pulp stones, particularly in more advanced types. In rare instances, *dens invaginatus* may co-occur with anomalies such as fusion or gemination, further complicating diagnosis and clinical management [3,15,39].

## 7. Advancements and Future Perspectives in the Management of *Dens Invaginatus*

Recent advancements have significantly improved the diagnosis and treatment of *dens invaginatus*, a condition that remains challenging due to its complex and variable anatomy. The adoption of cone beam computed tomography (CBCT) has enabled the precise three-dimensional assessment of invaginations and their relationship to surrounding anatomical structures. This technology has not only enhanced diagnostic accuracy but also supported the development of refined classification systems, such as the anatomical coding proposed by Ahmed and Dummer (2018) [21] and further validated by Mabrouk et al. (2021) [40], which facilitate more systematic documentation and case planning. In parallel, the integration of microsurgical techniques and the use of bioceramic materials have increased the success rate of treatment in anatomically complex cases [22]. Regenerative endodontic procedures now offer promising outcomes in immature teeth with necrotic pulps by promoting continued root development [41]. Looking ahead, innovations in artificial intelligence, genetics, and tissue engineering hold the potential to further enhance diagnostic precision and open new avenues for biologically based, minimally invasive therapies.

## 8. Final Remarks

The clinical significance of *dens invaginatus* lies in its predisposition to early pulpal and periapical pathology. The enamel-lined invagination, when exposed to the oral environment, creates a niche for bacterial colonization, particularly in cases where the internal enamel is hypomineralized or discontinuous. However, pulp necrosis may occur even in the absence of caries or trauma, as microorganisms can infiltrate through micro-communications or establish direct contact between the invagination and the periodontium. In conclusion, *dens invaginatus* is a complex and often underdiagnosed developmental anomaly with important clinical implications. Early detection is essential to guide appropriate management, which may range from preventive sealing and conservative restorative techniques to complex endodontic or surgical interventions, depending on the anatomical configuration and extent of pulpal involvement.

## Figures and Tables

**Table 1:** Summary of *dens invaginatus* classifications, relevance, and limitations.

Classification System	Key Feature	Usage	Relevance	Limitations
Hallet (1953) [16]	Clinical and radiographic appearance	Historical	Foundation for *dens invaginatus* taxonomy	Complex and has limited applicability
Oehlers (1957) [17]	Radiographic depth and communication	Widely adopted for clinical and research	Simple and easy radiographic application Correlates with clinical complexity Most relevant clinical classification	Limited to single description of invagination Lacks 3D anatomical variation
Ulmansky & Hermel (1964) [18]	Morphological variations and histology	Rarely used	Highlights spectrum of severity Emphasizes developmental and histological characteristics	Lacks radiographic criteria Less practical for diagnosis and treatment planning
Schulze & Brand (1972) [19]	Radiographic and clinical types	Research and academic focus	Covers full spectrum of morphological variations Useful for detailed anatomic documentation	No simple numerical system Complex for daily clinical use
Vincent-Townend (1974) [20]	Radiographic	Limited use	Useful in assessing severity and treatment planning Links morphology to clinical management	Not structured and lacks clinical details as it is reliant on radiographic appearance
Ahmed & Dummer (2018) [21]	Cone beam computed tomography and pulp morphology	Emerging	3D visualization. Identifies true pulp involvement Describes multiple invaginations	Coding system requires a learning curve High-resolution scans always required
Gul et al. (2021) [22]	Cone beam computed tomography	Emerging	3D visualization Enhanced Oehlers’ classification with additional Type IV proposed	High-resolution scans always required

**Table 2:** Overview of risks of pulpal involvement, clinical management, and prognosis of different types of *dens invaginatus*.

	Pulp/Periapical Involvement	Diagnostic Tool	Clinical Management	Prognosis and Follow Up
Type I	Minimal risk to pulp unless caries or bacterial ingress occurs	Periapical radiograph may be adequate	Preventive sealing of palatal pit or groove with resin-based materials Restoration if necessary Endodontic treatment only if secondary infection occurs	Good prognosis and early intervention help preserve pulp vitality Regular clinical and radiographic monitoring
Type II	Moderate to high risk of pulp involvement	CBCT recommended to assess depth and pulp communication	Preventive sealing or minimally invasive restoration If pulp compromised, vital pulp therapy or root canal treatment depending on health state of pulp	Good to fair prognosis if depth is within coronal third and detected early Radiographic follow-up needed to monitor healing
Type IIIa/IIIb	High risk of periapical/periodontal infection	CBCT essential to determine anatomy and extent	Selective treatment of infected invagination if pulp unaffected Often requires surgical access and debridement Orthograde or retrograde obturation with biocompatible materials Combined endodontic and periodontal approach in complex cases	Prognosis depends on anatomical complexity and extent of infection Long-term monitoring essential due to risk of reinfection

## Data Availability

The original contributions presented in this study are included in the article. Further inquiries can be directed to the corresponding author.
